# Crystal structure of death-associated protein kinase 1 in complex with the dietary compound resveratrol

**DOI:** 10.1107/S2052252520015614

**Published:** 2021-01-01

**Authors:** Takeshi Yokoyama, Ryoya Suzuki, Mineyuki Mizuguchi

**Affiliations:** a Faculty of Pharmaceutical Sciences, University of Toyama, 2630 Sugitani, Toyama, Toyama 930-0914, Japan

**Keywords:** DAPK1, resveratrol, Alzheimer’s disease, protein–drug interactions

## Abstract

Death-associated protein kinase 1 (DAPK1) is a large multidomain protein with an N-terminal serine/threonine protein kinase domain. DAPK1 is considered to be a promising molecular target for the treatment of Alzheimer’s disease (AD). In the present study, the inhibitory potency of resveratrol (RSV), a dietary polyphenol found in red wine, against the catalytic activity of DAPK1 was investigated. Kinetic and fluorescent probe competitive binding analyses revealed that RSV directly inhibited the catalytic activity of DAPK1 by binding to the ATP-binding site. Crystallographic analysis of DAPK1 in complex with RSV revealed that the A-ring of RSV occupied the nucleobase-binding position. Determination of the binding mode provided a structural basis for the design of more potent DAPK1 inhibitors. In conclusion, the data here clearly show that RSV is an ATP-competitive inhibitor of DAPK1, encouraging speculation that RSV may be useful for the development of AD inhibitors.

## Abstract 2

The dietary compound resveratrol is an inhibitor of death-associated protein kinase 1.

## 1. Introduction   

**Figure 1:**
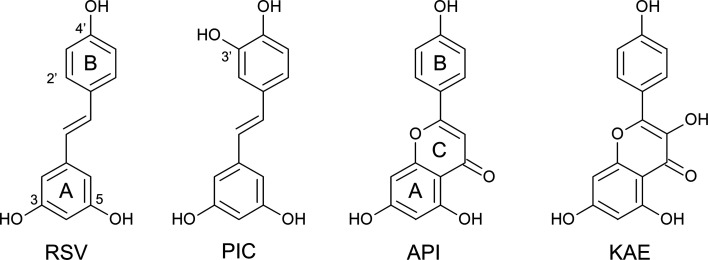
Chemical structures of RSV, PIC, API and KAE.

Resveratrol (RSV; 3,4′,5-trihydroxy-*trans*-stilbene; Fig. 1 ▸) is a natural polyphenolic phytoalexin found in grapes, peanuts, berry fruits and olives (Wang, Catana *et al.*, 2002 ▸). In particular, red wine contains a high concentration of RSV, ranging from 1 to 25 µ*M* depending on the grape varieties used (Gu *et al.*, 1999 ▸). RSV has attracted considerable attention owing to its cardioprotective activity, and has been suggested to be responsible for the ‘French paradox’, *i.e.* the inverse relationship between the consumption of red wine and cardiovascular disease rates in France (Renaud & de Lorgeril, 1992 ▸). RSV has also been shown to exhibit diverse biological effects including anti-inflammatory, antioxidant, neuroprotective and antitumor activities (Ashrafizadeh *et al.*, 2020 ▸; Banez *et al.*, 2020 ▸; Ahmadi & Ebrahimzadeh, 2020 ▸). Because cancer is a leading cause of death worldwide, the role of RSV in tumori­genesis has been extensively studied. The multistage chemical carcinogenesis involves disruption of intracellular signaling networks (Vineis *et al.*, 2010 ▸). Several studies have indicated that RSV directly or indirectly modulates signaling pathways, including the mitogen-activated protein kinase (MAPK) signaling pathway, the protein kinase C (PKC) signaling pathway and the phospho­inositide 3-kinase (PI3K)/protein kinase B (Akt) signaling pathway (Kundu & Surh, 2008 ▸). Although several protein kinases are associated with these pathways, only PKC isozymes and proto-oncogene serine/threonine-protein kinase (PIM1) have been shown to be specifically inhibited by RSV, and there is no structural information on the interactions between RSV and protein kinases (Slater *et al.*, 2003 ▸; Kim *et al.*, 2020 ▸).

Death-associated protein kinase 1 (DAPK1) is a Ca^2+^/calmodulin-regulated serine/threonine protein kinase (CaMK) composed of an N-terminal catalytic kinase domain, a Ca^2+^/calmodulin-binding domain, ankyrin repeats, an Roc/COR domain and a death domain. The multi-domain configuration of DAPK1 contributes to its interaction with various proteins, and thus DAPK1 functions as a central junction in various signaling pathways (Shiloh *et al.*, 2014 ▸). DAPK1 is known to be a regulator of apoptosis and autophagy, and to be a tumor suppressor protein (Deiss *et al.*, 1995 ▸; Inbal *et al.*, 2002 ▸). DAPK1 is also associated with the accumulation of amyloid-β and tau proteins in the brain in Alzheimer’s disease (AD). The expression of hippocampal DAPK1 was significantly increased in the brains of AD patients (Kim *et al.*, 2014 ▸). DAPK1 contributes to the pathogenesis of AD by the amyloidogenic processing of amyloid precursor protein and by phosphoryl­ating and stabilizing tau aggregates (Kim *et al.*, 2016 ▸; Farag & Roh, 2019 ▸; Kim *et al.*, 2014 ▸). It has been shown that gene knockout of DAPK1 ameliorated age-dependent neuro­degeneration in AD (Yukawa *et al.*, 2006 ▸). Increasing evidence indicates that DAPK1 is an important target molecule for the treatment of AD (Chico *et al.*, 2009 ▸). The inhibition of DAPK1 might also be an effective therapy for advanced endometrial adenocarcinomas and acute brain injury by hypoxia–ischemia, but the relevance of DAPK1 to those diseases is still under study (Tanaka *et al.*, 2010 ▸; Velentza *et al.*, 2003 ▸).

Feng and coworkers developed an ATP-competitive DAPK1 inhibitor with an octahedral ruthenium metal center which had an IC_50_ value against the phosphorylation activity of DAPK1 of 2.0 n*M* (Feng *et al.*, 2011 ▸). A pyrazolo­[3,4-*d*]pyrimidinone compound and an imidazopyramidazine compound have been reported to be DAPK1 inhibitors with dissociation constants (*K*_d_) of 0.3 and 0.24 µ*M*, respectively (Carlson *et al.*, 2013 ▸; Wilbek *et al.*, 2015 ▸). We have previously shown that natural flavonoids bind to the ATP-binding site of DAPK1 with various binding orientations (Yokoyama *et al.*, 2015 ▸). In addition, very recently we reported that purpurin, a natural anthraquinone found in the roots of *Rubia argyi*, is a potent DAPK1 inhibitor with a *K*_d_ value of 0.37 µ*M* (Yokoyama *et al.*, 2020 ▸). Although some DAPK1 inhibitors have been identified, as described above, none of them have reached the stage of clinical studies. The discovery and development of DAPK1 inhibitors is an ongoing project.

AD is a progressive neurodegenerative disorder associated with memory impairment, and thus continuous dietary intake of natural compounds that inhibit DAPK1 activity may be a good strategy to prevent the development of AD. RSV is known to be a safe material and is commercially available as a dietary supplement worldwide. Although our previous study using fluorescence spectroscopy suggested that RSV might directly interact with DAPK1 (Yokoyama *et al.*, 2015 ▸), there is no information about its inhibitory mechanism or binding modes. The investigation of interactions between DAPK1 and RSV may provide a novel strategy for the treatment of AD. In the present study, we tested the inhibitory activity of RSV against DAPK1. Kinetic analysis and a fluorescent probe competitive binding assay showed that RSV is an ATP-competitive DAPK1 inhibitor. Together with the results of crystallographic analysis of the DAPK1–RSV complex, we will discuss the binding of RSV to DAPK1.

## 2. Experimental procedure   

### 2.1. Protein production and chemicals   

The catalytic domains of the DAPK1 (residues 1–285) and ephrin type-A receptor 3 (EphA3; residues 613–905) proteins were prepared using an *Escherichia coli* system as described previously (Yokoyama *et al.*, 2020 ▸). The final protein samples were visualized by SDS–PAGE with Coomassie Brilliant Blue as a single band. In the case of DAPK1, successful crystallization assured the high purity of the protein. RSV, apigenin (API) and kaempferol (KAE) were purchased from Wako Pure Chemical Industries. Piceatannol (PIC) was purchased from Cayman Chemical Company. These chemicals were supplied at greater than 95% purity as determined by HPLC.

### 2.2. Intrinsic ATPase activity measurement   

The intrinsic ATPase activities of the kinases were measured by a colorimetric malachite green assay as described previously, with minor modifications (Sherwood *et al.*, 2013 ▸; Yokoyama *et al.*, 2019 ▸). The protein samples (0.1 µ*M* DAPK1 or 3 µ*M* EphA3) were incubated with the designated concentration of ATP at 298 K for 30–180 min. The reaction buffer consisted of 50 m*M* Tris–HCl pH 8.0, 100 m*M* NaCl, 10 m*M* MgCl_2_. The volume of the reaction buffer was 40 µl. To evaluate the effect of MES, 10 m*M* MES pH 8.0 was added. Because the resveratrol and the flavonoids were dissolved in dimethyl sulfoxide (DMSO), DMSO was added to the negative control samples at the same concentration. After the reaction period, 160 µl of malachite green reagent was added and the samples were incubated for 60 min or until the coloration stabilized. The malachite green reagent was made by mixing one volume of 4.2% ammonium molybdate in 4 *N* HCl with three volumes of 0.045%(*w*/*v*) malachite green supplemented with 0.01%(*v*/*v*) Tween 20. The absorbance at 620 nm was recorded in a 96-well clear microplate using a FilterMax F5 (Molecular Devices). The concentration of orthophos­phates was calculated by a calibration curve that was determined using NaH_2_PO_4_. The reaction velocities and standard errors were calculated from at least quintuplicate independent experiments.

### 2.3. ANS competitive binding assay   

The ANS competitive assay was carried out as described previously, with some modifications (Yokoyama *et al.*, 2015 ▸, 2020 ▸). Samples containing 2 µ*M* DAPK1, 50 m*M* Tris–HCl pH 8, 100 m*M* NaCl, 0–300 µ*M* ANS and 0–100 µ*M* RSV were prepared in a 96-well black microplate and incubated for 60 min at room temperature. The volume of each sample was 200 µl. After the incubation period, the fluorescence intensity was measured using a GENios (Tecan Group) with excitation and emission wavelengths of 360 and 465 nm, respectively. The half-maximal bound concentration (BC_50_) values were calculated by fitting to a four-parameter logistic model using the least-squares method (Sebaugh, 2011 ▸). The BC_50_ values and standard errors were calculated from quadruplicate independent experiments.

### 2.4. Protein crystallography   

DAPK1 crystals were grown by the hanging-drop vapor-diffusion method at 293 K. Prior to crystallization, 16 mg ml^−1^ DAPK1 and 20 m*M* RSV or PIC in DMSO were mixed in a ratio of 9:1 and the mixtures were incubated at room temperature for 60 min. Crystals of DAPK1–REV were grown by equilibration of a droplet composed of 2 µl protein sample and 2 µl reservoir solution (2.4 *M* ammonium sulfate, 0.1 *M* Tris–HCl pH 8.0) against 1 ml reservoir solution. The reservoir conditions for the DAPK–PIC and DAPK1–RSV–MES complexes were 2.0 *M* ammonium sulfate, 0.1 *M* Tris–HCl pH 7.0 and 1.8 *M* ammonium sulfate, 0.1 *M* MES pH 6.5, respectively. Crystals began to appear within three days and the crystal growth was stopped after one week. The crystals were directly cooled in liquid nitrogen until the X-ray diffraction experiments.

Cryogenic X-ray diffraction data were collected on beamline BL-17A at the Photon Factory (PF) and on beamline NW12A at the Photon Factory Advanced Ring (PF-AR) in Japan. The diffraction data sets were integrated using the *XDS* program package (Kabsch, 2010 ▸). We adopted an *I*/σ(*I*) cutoff of 2.0 in the highest resolution shells. Because the crystals in the present study were isomorphous to those of a previous DAPK1–inhibitor complex (PDB code 5aux), structure refinement could be directly started using the coordinates of the DAPK1–inhibitor complex without performing Patterson-based molecular replacement (Yokoyama *et al.*, 2015 ▸). Prior to refinement, the coordinates of the inhibitor were omitted. The 3D structures and dictionary data for RSV and PIC were generated using the *PRODRG* server (Schüttelkopf & van Aalten, 2004 ▸). The protein structures were refined using *phenix.refine* with stepwise cycles of manual model building using *Coot* (Liebschner *et al.*, 2019 ▸; Emsley *et al.*, 2010 ▸). The final models were evaluated using the Protein Data Bank validation suite (Yang *et al.*, 2004 ▸). All structural figures were created using *PyMOL* (Schrödinger).

## 3. Results   

### 3.1. Intrinsic ATPase activity of DAPK1   

**Figure 2:**
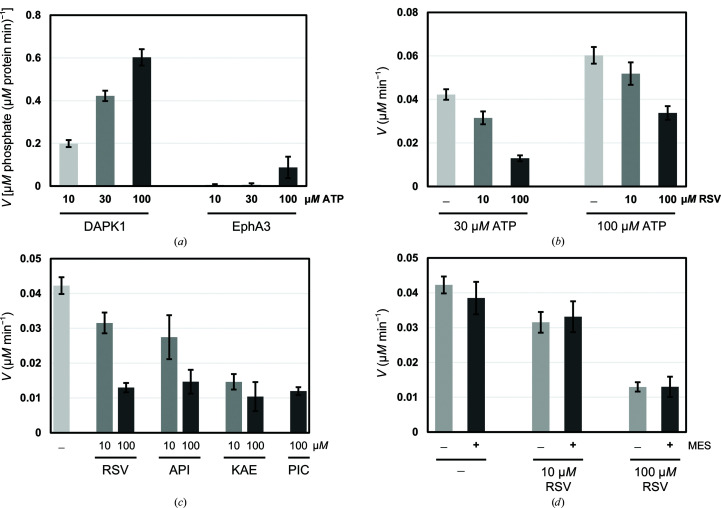
(*a*) DAPK1 exhibited intrinsic ATPase activity. The histogram shows the reaction rates for the ATPase activities of DAPK1 and EphA3 at 10, 30 and 100 µ*M* ATP. The reaction rates were obtained by dividing the product concentration by the protein concentration and the reaction time. (*b*) RSV inhibited the ATPase activity of DAPK1 in a dose-dependent manner at 30 and 100 µ*M* ATP. The reaction rates were obtained by dividing the product concentration by the reaction time. (*c*) The histograms show the inhibitory effect of RSV-related compounds against the intrinsic ATPase activity of DAPK1 at 30 µ*M* ATP. (*d*) The presence of 10 m*M* MES does not influence the reaction rate either in the presence or the absence of RSV.

We assessed the intrinsic ATP hydrolysis (ATPase) activity of the catalytic domain of DAPK1 (residues 1–285). The ATPase activity was evaluated using the classic malachite green assay (Sherwood *et al.*, 2013 ▸), in which orthophosphates, which are ATP hydrolysis products, are detected. We used tyrosine kinase ephrin type-A receptor 3 (EphA3) as a control. The ATPase activities of DAPK1 and EphA3 were clearly observed and increased in an ATP concentration-dependent manner, indicating that the ATP molecules were hydrolyzed by the kinases [Fig. 2 ▸(*a*)]. The intrinsic ATPase activity of EphA3 was remarkably lower in comparison to that of DAPK1. While the ATPase activity of DAPK1 could be accurately detected at a protein concentration of lower than 0.1 µ*M*, the ATPase activity of EphA3 was only detectable at a protein concentration of higher than 1 µ*M*. In general, the activity of protein kinases is also easily detected using a protein kinase assay kit. However, with regard to the inhibition assay, there is the possibility of a false-positive result from the binding of inhibitors to components of the kits. It has been reported that measuring the intrinsic ATPase activity is a more reliable way of identifying an interleukin-2-inducible T-cell kinase inhibitor than measuring the phosphorylation-transfer activity (Kashem *et al.*, 2007 ▸). Therefore, we preferred the use of a simple malachite green assay to investigate the inhibitory activity of compounds in this study.

### 3.2. RSV inhibited the intrinsic ATPase activity of DAPK1   

The hydrolysis velocity at a concentration of 0.1 µ*M* DAPK1 and 30 µ*M* ATP was 0.042 µ*M* min^−1^. The addition of 10 and 100 µ*M* RSV significantly reduced the hydrolysis velocity to 0.031 and 0.013 µ*M* min^−1^, respectively [Fig. 2 ▸(*b*)]. Similarly, the hydrolysis velocity at 100 µ*M* ATP was 0.06 µ*M* min^−1^ in the absence of RSV, and the addition of 10 and 100 µ*M* RSV significantly decreased the hydrolysis velocity to 0.052 and 0.034 µ*M* min^−1^, respectively. These results clearly indicate that RSV exhibits inhibitory activity against the ATPase activity of DAPK1.

**Figure 3:**
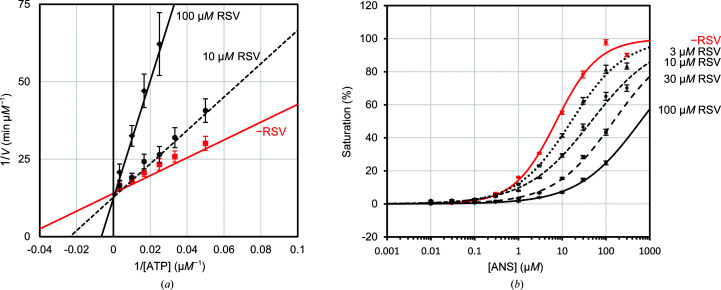
(*a*) The Lineweaver–Burk plot for inhibition of the ATPase activity by RSV demonstrates a large effect on *K*_m_ and no effect on *K*_cat_. Regression lines are drawn for the mean of at least five experiments. (*b*) Semi-log plot for ANS competitive binding assays in the absence of RSV (red continuous line) and in the presence of 3 µ*M* RSV (black dotted line), 10 µ*M* RSV (black short-dashed line), 30 µ*M* RSV (black long-dashed line) and 10 µ*M* RSV (black continuous line). RSV decreased the BC_50_ values in a dose-dependent manner.

In order to determine the type of inhibition by RSV, the enzymatic kinetics in the absence and presence of RSV were analyzed by means of a Lineweaver–Burk plot. While the addition of 10 and 100 µ*M* RSV did not have any effect on the *K*_cat_ values, the *K*_m_ values were significantly increased in an RSV concentration-dependent manner, indicating that the inhibition kinetics of RSV correspond to an ATP competitive inhibition model [Fig. 3 ▸(*a*) and Table 1 ▸]. For further verification, a competitive binding assay was performed using the fluorescence probe 8-anilino­naphthalene-1-sulfonic acid (ANS). We have previously reported that ANS specifically binds to the ATP-binding site of DAPK1, and the fluorescence emission of ANS was increased by binding to DAPK1 (Yokoyama *et al.*, 2015 ▸). Therefore, ANS is a useful tool for the discovery of ATP-competitive DAPK1 inhibitors. In this assay, the fractions of ANS-saturated DAPK1 at various ANS concentrations in the presence or absence of the compounds were measured and the half-maximal bound concentration (BC_50_) values were estimated by fitting to a four-parameter logistic model (Sebaugh, 2011 ▸). The BC_50_ value in the absence of RSV was 7.3 µ*M* and the BC_50_ values in the presence of 3, 10, 30 and 100 µ*M* RSV were 16, 46, 150 and 600 µ*M*, respectively [Fig. 3 ▸(*b*)]. RSV clearly inhibited the binding of ANS to DAPK1 in an RSV concentration-dependent manner and 100 µ*M* RSV almost completely inhibited the binding of ANS. The ANS competitive assay also showed that RSV is an ATP-competitive DAPK1 inhibitor.

In addition, we tested the inhibitory activity of API, KAE and PIC against the intrinsic ATPase activity of DAPK1 (Fig. 1 ▸). API and KAE are natural flavonoids that are structurally related to RSV and have been shown to bind to the ATP-binding site of DAPK1. The inhibitory potency of API was similar to that of RSV [Fig. 2 ▸(*c*)]. The inhibitory potency of KAE was higher than that of API, which was in agreement with a previous ANS competitive binding assay (Yokoyama *et al.*, 2015 ▸). PIC is a 3-hydroxy RSV derivative (Fig. 1 ▸). PIC also inhibited the ATPase activity of DAPK1.

### 3.3. Crystal structure of DAPK1 in complex with RSV and PIC   

**Figure 4:**
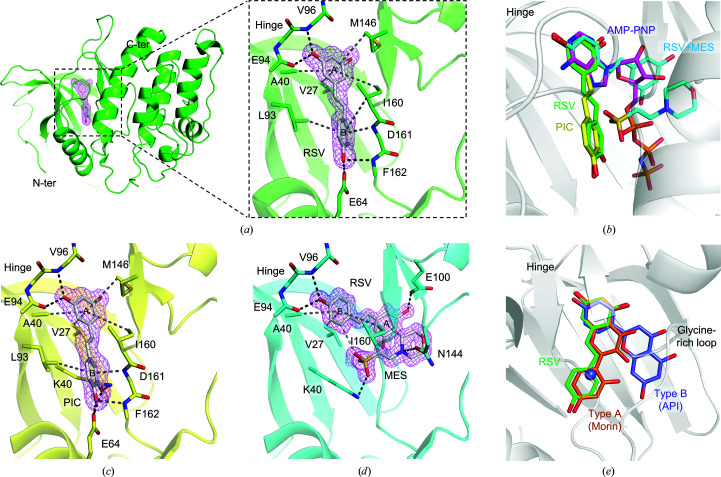
(*a*) Overall structure of DAPK1 in complex with RSV and close-up view of the binding of RSV to the ATP-binding site (PDB entry 7ccu). The magenta mesh indicates the polder map (Liebschner *et al.*, 2017 ▸) for RSV contoured at 3.3σ. The black and gray dashed lines indicate hydrogen bonds and CH⋯π interactions, respectively. (*b*) Comparison of the binding modes of AMP-PNP (PDB entry 1jkk), RES (PDB entry 7ccu), PIC (PDB entry 7ccv) and RES+MES (PDB entry 7ccw). The C atoms of AMP-PNP, RSV, PIC and RSV+MES are colored purple, green, yellow and cyan, respectively. (*b*) The binding mode of PIC (PDB entry 7ccv). The polder map was contoured at 3.3σ and is shown as a magenta mesh. (*d*) Simultaneous binding of RES and MES (PDB entry 7ccw). The polder map was contoured at 3.3σ and is shown as a magenta mesh. (*e*) Comparison of the binding modes of RSV (PDB entry 7ccu), API (PDB entry 5auv) and morin (PDB entry 5auy) in their DAPK1 complexes. The C atoms of RSV, API and morin are colored green, slate blue and orange, respectively.

Information on the atomic interactions between DAPK1 and RSV will provide a better understanding of the inhibition mechanism and better ideas for the development of potent DAPK1 inhibitors. The crystal structure of DAPK1 in complex with RSV was solved at 1.65 Å resolution (Table 2 ▸). The overall structure of DAPK1–RSV was almost identical to those of the apo form (PDB entry 1jks; Tereshko *et al.*, 2001 ▸) and the AMP-PNP complex (PDB entry 1jkk; Tereshko *et al.*, 2001 ▸), with an r.m.s.d. of 0.18 Å. It was found that RSV bound to the ATP-binding site, in agreement with kinetic analysis and the ANS competitive assay: distinctive electron density was observed in the ATP-binding site and its shape corresponded to the molecular shape of RSV [Fig. 4 ▸(*a*)]. The A-ring of RSV occupied the binding site of the adenyl base of ATP and the 3-hydroxy group was hydrogen-bonded to the carbonyl group of Glu94 and the amide group of Val96 in the hinge region [Figs. 4 ▸(*a*) and 4 ▸(*b*)]. The A-ring was also stabilized by several C—H⋯π interactions with the side chains of Val27, Ala40, Met146 and Ile160. The B-ring was located deep in the hydrophobic pocket, forming a hydrogen bond to Glu64, C—­H⋯π interactions with Leu93 and Ile160, and NH⋯π interactions with the amido group of Asp161. The crystal structure of DAPK1 in complex with PIC was also solved at 1.75 Å resolution. The overall structure of the PIC complex was similar to that of the RSV complex, with an r.m.s.d. of 0.18 Å. The PIC molecule was clearly observed in the ATP-binding site and the binding mode of PIC was almost identical to that of RSV [Fig. 4 ▸(*c*)]. In comparison to the RSV complex, additional hydrogen bonds were formed between the 3′-hydroxy group of PIC, Glu64 and Lys42. These hydrogen bonds may increase the binding affinity for DAPK1.

After we optimized the crystallization conditions for the DAPK1–RSV complex, we found that the crystal quality was significantly improved by using MES pH 6.5. We therefore solved the structure of a crystal obtained using MES buffer pH 6.5 at 1.4 Å resolution. While the overall structure was quite similar to that of the single RSV complex, with an r.m.s.d. of 0.16 Å, the RSV and MES molecules were observed in the ATP-binding site and the binding mode of RSV was different from that of single RSV [Figs. 4 ▸(*b*) and 4( ▸ *d*)]. The B-ring of RSV was located in the hinge region, and the 4′-hydroxy group was hydrogen-bonded to the carbonyl group of Glu94 and the amide group of Val96. The A-ring was located in the ribose site and the 3-hydroxy group was hydrogen-bonded to Glu100. The morpholino ring of MES was stacked on the A-ring of RSV by C—H⋯π interactions, and the N atom of the morpholino ring formed a hydrogen bond to Asn144. The sulfate group of MES occupied the position of the α-phosphate group of AMP-PNP. The sulfate group formed a salt bridge with Lys42. The C—H⋯O hydrogen bond between the 2′-H of RSV and the sulfate group of MES may also contribute to stabilization of the binding of MES. The simultaneous binding of RSV and MES implies that MES might synergistically inhibit the ATPase activity of DAPK1 with RSV or might increase the inhibitory potency of RSV. However, the addition of 10 m*M* MES to the ATPase activity assay did not have a significant effect on the inhibitory potency of RSV [Fig. 2 ▸(*d*)]. The presence of MES may not increase the binding affinity of RSV to DAPK1. However, from the viewpoint of the development of DAPK1 inhibitors, structural information on the simultaneous binding of RSV and MES would be useful for fragment-based drug discovery. Hybridization of RSV and MES may increase the inhibitory potency.

## 4. Discussion and conclusion   

The ATPase activity of protein kinases is a hydrolysis reaction of ATP to ADP and orthophosphate using a water molecule as a nucleophile instead of a protein substrate. Therefore, it is conceivable that the ATPase activity is strongly dependent on the environment of the nucleophilic water molecule located close to the catalytic residues (Lys42 and Glu64 in DAPK1). Although the protein kinase activity (phosphorylation activity) has been broadly investigated, there are a few protein kinases that have been shown to exhibit intrinsic ATPase activity. To date, the ATPase activities of mitogen-activated protein/ERK kinase kinase 2 (MEKK2), MEK1, protein kinase C (PKC), dual-specificity protein kinase 2 (ERK2), JNK3, p38γ and p38α have been identified (Fox *et al.*, 1999 ▸; Rominger *et al.*, 2007 ▸; Ahmad *et al.*, 2013 ▸). MEKK2 and MEK1 are classified into the STE protein kinase subfamily while ERK2, JNK3, p38α and p38γ are classified into the CMGC subfamily. Our data clearly showed that DAPK1, which is classified into the CaMK subfamily, possesses ATPase activity, but the ATPase activity of EphA3, which is classified into the TK subfamily, was drastically lowered. Intrinsic ATPase activity may be widely present in serine/threonine protein kinases but not in tyrosine kinases.

Although RSV is known to exhibit diverse biological effects, research into the interactions between RSV and protein kinases is still at an early stage. To the best of our knowledge, only a few protein kinases are known to interact directly with RSV, and RSV inhibited the ATPase activity of purified PKC and its isozymes (Slater *et al.*, 2003 ▸; Yoon *et al.*, 2002 ▸; Haworth & Avkiran, 2001 ▸). Recently, it was reported that RSV binds to PIM1 in a pull-down-based assay (Kim *et al.*, 2020 ▸). However, the detailed inhibitory mechanism has not been elucidated owing to a lack of co-crystal structures of the kinases and RSV. Here, a comprehensive analysis using Michaelis–Menten kinetics, an ANS competitive binding assay and protein crystallography clearly showed that RSV is an ATP-competitive inhibitor of DAPK1.

RSV is composed of two aromatic phenyl rings with three hydroxy groups, and thus it is conceivable that RSV can bind to a highly hydrophobic pocket and also form hydrogen bonds to polar groups (Saqib *et al.*, 2018 ▸). It is therefore reasonable that the ATP-binding site can accept the binding of RSV, because the adenyl group also possesses an aromatic property and hydrogen-bond donors and acceptors. Indeed, the DAPK1–RSV crystal structures showed that the A-ring or B-ring of RSV occupy the hinge region to which the adenyl group of AMP-PNP binds [Fig. 4 ▸(*b*)]. RSV is able to bind to various proteins ranging from ligases to oxidoreductases and hydrolases (Sajish & Schimmel, 2015 ▸; Buryanovskyy *et al.*, 2004 ▸; Shukla *et al.*, 2015 ▸). However, there are no significant similarities in the sequences and structures of the binding pockets of these proteins (Saqib *et al.*, 2018 ▸). The bindability of nucleobases may be a good predictor of the binding of RSV.

We have previously reported natural flavonoids to be DAPK1 inhibitors with two distinctive binding modes referred to as type A and type B (Yokoyama *et al.*, 2015 ▸). The binding modes of API and KAE are type B binding modes. In the type B binding mode, binding of the flavonoids is associated with the binding of chloride ion. While the B-ring of API occupies the hinge region, the AC-ring points to the glycine-rich loop [Fig. 4 ▸(*e*)]. Based on the structural similarity among RSV, API and KAE, we initially speculated that the binding mode of RSV would be similar to those of API and KAE. However, we found that the binding mode of RSV corresponded to the type A binding mode, in which the B-ring is turned towards the side chain of Glu64 and the AC-ring occupies the hinge region [Fig. 4 ▸(*e*)]. The binding of API and KAE was stabilized by interaction of the central pyran ring (C-ring) with the glycine-rich loop and the chloride ion. The lack of a pyran ring may force RSV to bind in the type A mode. In terms of the structure–activity relationship study, determination of the binding orientation would be helpful for the design of RSV derivatives with improved inhibitory activity: an optimization of 2′-H to interact with the glycine-rich loop would increase the binding affinity to DAPK1.

RSV is known to be very safe and well tolerated. After oral dosage with RSV, it is extensively metabolized in the intestine and liver, and is rapidly eliminated (Walle *et al.*, 2004 ▸). Therefore, a major concern regarding the clinical use of RSV is its poor bioavailability. However, this poor bioavailability is currently being overcome by several approaches, including nanoencapsulation of RSV and the development of long-lived RSV derivatives (Sergides *et al.*, 2016 ▸; Chimento *et al.*, 2019 ▸). It should be noted that several animal studies have indicated that resveratrol is blood–brain barrier (BBB) permeable (Wang, Xu *et al.*, 2002 ▸; Frozza *et al.*, 2013 ▸). Because BBB permeability is critical for the development of neurodegenerative disorder inhibitors, BBB permeability of RSV would be a great advantage. The increasing number of studies designed to improve the oral bioavailability of RSV encourages us to expect that RSV and RSV derivatives could be useful for the treatment of AD in the future.

In summary, we have demonstrated that RSV inhibits the intrinsic ATPase activity of DAPK1 by binding to the ATP-binding site. Crystallographic analysis of the DAPK1–RSV complex revealed that the binding of RSV to DAPK1 was stabilized by occupation of the A-ring at the nucleobase position and by hydrogen bonds to the hinge region. Determination of the binding orientation provided key information for improvement of the binding affinity of RSV to DAPK1. Although the development of RSV as a clinical drug is still under way, the present data encourage further development of RSV for the treatment of DAPK1-associated diseases.

## Supplementary Material

PDB reference: death-associated protein kinase 1, complex with resveratrol, 7ccu

PDB reference: complex with piceatannol, 7ccv

PDB reference: complex with resveratrol and MES, 7ccw

## Figures and Tables

**Table 1:** Kinetic parameters for the intrinsic ATPase activity of DAPK1 in the absence and presence of RSV

	DAPK1	DAPK1 + 10 µ*M* RSV	DAPK1 + 100 µ*M* RSV
*K*_m_ (µ*M*)	21 ± 2.4	42 ± 10	150 ± 30
*K*_cat_ (min^−1^)	0.72 ± 0.0051	0.79 ± 0.12	0.81 ± 0.10
*K*_cat_/*K*_m_ (µ*M* ^−1^ min^−1^)	0.044 ± 0.0043	0.024 ± 0.0032	0.0066 ± 0.00090
Coefficient of determination (*R* ^2^)	0.995	0.993	0.999

**Table 2:** X-ray diffraction data-collection and refinement statistics

	DAPK1–RSV	DAPK1–PIC	DAPK1–RSV–MES
Crystal data			
Beamline	BL-17A, PF	NW12A, PF-AR	BL-17A, PF
Resolution range (Å)	41.4–1.65 (1.71–1.65)	41.3–1.75 (1.82–1.75)	41.3–1.40 (1.45–1.40)
Space group	*P*2_1_2_1_2_1_	*P*2_1_2_1_2_1_	*P*2_1_2_1_2_1_
*a*, *b*, *c* (Å)	46.9, 62.2, 88.2	46.8, 61.7, 87.5	46.8, 62.3, 88.3
Observed reflections	209947 (21180)	107637 (4668)	268272 (17428)
Unique reflections	30997 (2999)	24148 (1551)	51220 (4865)
Completeness (%)	97.3 (95.5)	92.5 (60.8)	99.3 (96.0)
*R*_meas_ (%)	4.9 (57.8)	5.1 (52.5)	3.5 (53.7)
*R*_p.i.m._ (%)	1.9 (21.5)	2.3 (29.2)	1.5 (27.1)
Multiplicity	6.8 (7.1)	4.5 (3.0)	5.2 (3.6)
〈*I*/σ(*I*)〉	24.1 (3.5)	20.4 (2.5)	27.3 (2.5)
CC_1/2_	0.999 (0.881)	0.999 (0.781)	1.000 (0.805)
Refinement data			
*R*/*R*_free_ (%)	17.5/19.9	17.3/20.8	17/18.8
R.m.s.d., bonds (Å)	0.006	0.006	0.006
R.m.s.d., angles (°)	0.86	0.86	0.91
Average *B* factor (Å^2^)			
Overall	24.8	24.5	20.1
Protein	23.9	23.7	18.8
Ligand	25.4	23.5	19.3
Water	33.1	31.9	30
MES	—	—	20.2
Ramachandran plot (%)			
Favored	97.1	96.3	97.5
Allowed	2.5	3.7	2.2
PDB code	7ccu	7ccv	7ccw

## References

[bb2] Ahmad, S., Hughes, M. A., Johnson, G. L. & Scott, J. E. (2013). *J. Biomol. Screen.* **18**, 388–399. 10.1177/1087057112466430 PMC3723327 23134735

[bb3] Ahmadi, R. & Ebrahimzadeh, M. A. (2020). *Eur. J. Med. Chem.* **200**, 112356. 10.1016/j.ejmech.2020.112356 32485531

[bb4] Ashrafizadeh, M., Zarrabi, A., Najafi, M., Samarghandian, S., Mohammadinejad, R. & Ahn, K. S. (2020). *Phytother. Res.* **34**, 2867–2888. 10.1002/ptr.6732 32491273

[bb5] Banez, M. J., Geluz, M. I., Chandra, A., Hamdan, T., Biswas, O. S., Bryan, N. S. & Von Schwarz, E. R. (2020). *Nutr. Res.* **78**, 11–26. 10.1016/j.nutres.2020.03.002 32428778

[bb6] Buryanovskyy, L., Fu, Y., Boyd, M., Ma, Y., Hsieh, T.-C., Wu, J. M. & Zhang, Z. (2004). *Biochemistry*, **43**, 11417–11426. 10.1021/bi049162o PMC3650734 15350128

[bb7] Carlson, D. A., Franke, A. S., Weitzel, D. H., Speer, B. L., Hughes, P. F., Hagerty, L., Fortner, C. N., Veal, J. M., Barta, T. E., Zieba, B. J., Somlyo, A. V., Sutherland, C., Deng, J. T., Walsh, M. P., MacDonald, J. A. & Haystead, T. A. J. (2013). *ACS Chem. Biol.* **8**, 2715–2723. 10.1021/cb400407c PMC4445880 24070067

[bb8] Chico, L. K., Van Eldik, L. J. & Watterson, D. M. (2009). *Nat. Rev. Drug Discov.* **8**, 892–909. 10.1038/nrd2999 PMC2825114 19876042

[bb9] Chimento, A., De Amicis, F., Sirianni, R., Sinicropi, M., Puoci, F., Casaburi, I., Saturnino, C. & Pezzi, V. (2019). *Int. J. Mol. Sci.* **20**, 1381. 10.3390/ijms20061381 PMC6471659 30893846

[bb10] Deiss, L. P., Feinstein, E., Berissi, H., Cohen, O. & Kimchi, A. (1995). *Genes Dev.* **9**, 15–30. 10.1101/gad.9.1.15 7828849

[bb11] Emsley, P., Lohkamp, B., Scott, W. G. & Cowtan, K. (2010). *Acta Cryst.* D**66**, 486–501. 10.1107/S0907444910007493 PMC2852313 20383002

[bb12] Farag, A. K. & Roh, E. J. (2019). *Med. Res. Rev.* **39**, 349–385. 10.1002/med.21518 29949198

[bb13] Feng, L., Geisselbrecht, Y., Blanck, S., Wilbuer, A., Atilla-Gokcumen, G. E., Filippakopoulos, P., Kräling, K., Celik, M. A., Harms, K., Maksimoska, J., Marmorstein, R., Frenking, G., Knapp, S., Essen, L.-O. & Meggers, E. (2011). *J. Am. Chem. Soc.* **133**, 5976–5986. 10.1021/ja1112996 PMC3076536 21446733

[bb14] Fox, T., Fitzgibbon, M. J., Fleming, M. A., Hsiao, H.-M., Brummel, C. L. & Su, M. S.-S. (1999). *FEBS Lett.* **461**, 323–328. 10.1016/s0014-5793(99)01488-x 10567720

[bb15] Frozza, R. L., Bernardi, A., Hoppe, J. B., Meneghetti, A. B., Matté, A., Battastini, A. M., Pohlmann, A. R., Guterres, S. S. & Salbego, C. (2013). *Mol. Neurobiol.* **47**, 1066–1080. 10.1007/s12035-013-8401-2 23315270

[bb16] Gu, X., Creasy, L., Kester, A. & Zeece, M. (1999). *J. Agric. Food Chem.* **47**, 3223–3227. 10.1021/jf981211e 10552635

[bb17] Haworth, R. S. & Avkiran, M. (2001). *Biochem. Pharmacol.* **62**, 1647–1651. 10.1016/s0006-2952(01)00807-3 11755118

[bb18] Inbal, B., Bialik, S., Sabanay, I., Shani, G. & Kimchi, A. (2002). *J. Cell Biol.* **157**, 455–468. 10.1083/jcb.200109094 PMC2173279 11980920

[bb19] Kabsch, W. (2010). *Acta Cryst.* D**66**, 125–132. 10.1107/S0907444909047337 PMC2815665 20124692

[bb20] Kashem, M. A., Nelson, R. M., Yingling, J. D., Pullen, S. S., Prokopowicz, A. S., Jones, J. W., Wolak, J. P., Rogers, G. R., Morelock, M. M., Snow, R. J., Homon, C. A. & Jakes, S. (2007). *J. Biomol. Screen.* **12**, 70–83. 10.1177/1087057106296047 17166826

[bb21] Kim, B. M., You, M.-H., Chen, C.-H., Lee, S., Hong, Y., Kimchi, A., Zhou, X. Z. & Lee, T. H. (2014). *Cell Death Dis.* **5**, e1237. 10.1038/cddis.2014.216 PMC4047864 24853415

[bb22] Kim, B. M., You, M.-H., Chen, C.-H., Suh, J., Tanzi, R. E. & Lee, T. H. (2016). *Hum. Mol. Genet.* **25**, 2498–2513. 10.1093/hmg/ddw114 PMC6086563 27094130

[bb23] Kim, S., Kim, W., Kim, D.-H., Jang, J.-H., Kim, S.-J., Park, S.-A., Hahn, H., Han, B. W., Na, H.-K., Chun, K.-S., Choi, B. Y. & Surh, Y.-J. (2020). *Arch. Biochem. Biophys.* **689**, 108413. 10.1016/j.abb.2020.108413 32473133

[bb24] Kundu, J. K. & Surh, Y.-J. (2008). *Cancer Lett.* **269**, 243–261. 10.1016/j.canlet.2008.03.057 18550275

[bb1] Liebschner, D., Afonine, P. V., Baker, M. L., Bunkóczi, G., Chen, V. B., Croll, T. I., Hintze, B., Hung, L.-W., Jain, S., McCoy, A. J., Moriarty, N. W., Oeffner, R. D., Poon, B. K., Prisant, M. G., Read, R. J., Richardson, J. S., Richardson, D. C., Sammito, M. D., Sobolev, O. V., Stockwell, D. H., Terwilliger, T. C., Urzhumtsev, A. G., Videau, L. L., Williams, C. J. & Adams, P. D. (2019). *Acta Cryst.* D**75**, 861–877. 10.1107/S2059798319011471 PMC6778852 31588918

[bb55] Liebschner, D., Afonine, P. V., Moriarty, N. W., Poon, B. K., Sobolev, O. V., Terwilliger, T. C. & Adams, P. D. (2017). *Acta Cryst.* D**73**, 148–157. 10.1107/S2059798316018210 PMC5297918 28177311

[bb25] Renaud, S. & de Lorgeril, M. (1992). *Lancet*, **339**, 1523–1526. 10.1016/0140-6736(92)91277-f 1351198

[bb26] Rominger, C. M., Schaber, M. D., Yang, J., Gontarek, R. R., Weaver, K. L., Broderick, T., Carter, L., Copeland, R. A. & May, E. W. (2007). *Arch. Biochem. Biophys.* **464**, 130–137. 10.1016/j.abb.2007.04.004 17490600

[bb27] Sajish, M. & Schimmel, P. (2015). *Nature*, **519**, 370–373. 10.1038/nature14028 PMC4368482 25533949

[bb28] Saqib, U., Kelley, T. T., Panguluri, S. K., Liu, D., Savai, R., Baig, M. S. & Schürer, S. C. (2018). *Front. Pharmacol.* **9**, 1201. 10.3389/fphar.2018.01201 PMC6207623 30405416

[bb30] Schüttelkopf, A. W. & van Aalten, D. M. F. (2004). *Acta Cryst.* D**60**, 1355–1363. 10.1107/S0907444904011679 15272157

[bb31] Sebaugh, J. L. (2011). *Pharm. Stat.* **10**, 128–134. 10.1002/pst.426 22328315

[bb32] Sergides, C., Chirilă, M., Silvestro, L., Pitta, D. & Pittas, A. (2016). *Exp. Ther. Med.* **11**, 164–170. 10.3892/etm.2015.2895 PMC4726856 26889234

[bb33] Sherwood, A. R., Paasch, B. C., Worby, C. A. & Gentry, M. S. (2013). *Anal. Biochem.* **435**, 54–56. 10.1016/j.ab.2012.10.044 PMC3577954 23201267

[bb34] Shiloh, R., Bialik, S. & Kimchi, A. (2014). *Apoptosis*, **19**, 286–297. 10.1007/s10495-013-0924-5 24220854

[bb35] Shukla, P. K., Gautam, L., Sinha, M., Kaur, P., Sharma, S. & Singh, T. P. (2015). *Biochim. Biophys. Acta*, **1854**, 269–277. 10.1016/j.bbapap.2014.12.017 25541253

[bb36] Slater, S. J., Seiz, J. L., Cook, A. C., Stagliano, B. A. & Buzas, C. J. (2003). *Biochim. Biophys. Acta*, **1637**, 59–69. 10.1016/s0925-4439(02)00214-4 12527408

[bb37] Tanaka, T., Bai, T. & Yukawa, K. (2010). *Int. J. Oncol.* **37**, 1017–1022. 10.3892/ijo_00000753 20811724

[bb77] Tereshko, V., Teplova, M., Brunzelle, J., Watterson, D. M. & Egli, M. (2001). *Nat. Struct. Biol.* **8**, 899–907. 10.1038/nsb1001-899 11573098

[bb38] Velentza, A. V., Wainwright, M. S., Zasadzki, M., Mirzoeva, S., Schumacher, A. M., Haiech, J., Focia, P. J., Egli, M. & Watterson, D. M. (2003). *Bioorg. Med. Chem. Lett.* **13**, 3465–3470. 10.1016/s0960-894x(03)00733-9 14505650

[bb39] Vineis, P., Schatzkin, A. & Potter, J. D. (2010). *Carcinogenesis*, **31**, 1703–1709. 10.1093/carcin/bgq087 PMC3025741 20430846

[bb40] Walle, T., Hsieh, F., DeLegge, M. H., Oatis, J. E. Jr & Walle, U. K. (2004). *Drug Metab. Dispos.* **32**, 1377–1382. 10.1124/dmd.104.000885 15333514

[bb41] Wang, Q., Xu, J., Rottinghaus, G. E., Simonyi, A., Lubahn, D., Sun, G. Y. & Sun, A. Y. (2002). *Brain Res.* **958**, 439–447. 10.1016/s0006-8993(02)03543-6 12470882

[bb42] Wang, Y., Catana, F., Yang, Y., Roderick, R. & van Breemen, R. B. (2002). *J. Agric. Food Chem.* **50**, 431–435. 10.1021/jf010812u 11804508

[bb43] Wilbek, T. S., Skovgaard, T., Sorrell, F. J., Knapp, S., Berthelsen, J. & Strømgaard, K. (2015). *ChemBioChem*, **16**, 59–63. 10.1002/cbic.201402512 PMC4431585 25382253

[bb44] Yang, H., Guranovic, V., Dutta, S., Feng, Z., Berman, H. M. & Westbrook, J. D. (2004). *Acta Cryst.* D**60**, 1833–1839. 10.1107/S0907444904019419 15388930

[bb45] Yokoyama, T., Kitakami, R. & Mizuguchi, M. (2019). *Eur. J. Med. Chem.* **167**, 153–160. 10.1016/j.ejmech.2019.02.011 30771603

[bb46] Yokoyama, T., Kosaka, Y. & Mizuguchi, M. (2015). *J. Med. Chem.* **58**, 7400–7408. 10.1021/acs.jmedchem.5b00893 26322379

[bb47] Yokoyama, T., Wijaya, P., Kosaka, Y. & Mizuguchi, M. (2020). *Acta Cryst.* D**76**, 438–446. 10.1107/S2059798320003940 32355040

[bb48] Yoon, S.-H., Kim, Y.-S., Ghim, S.-Y., Song, B.-H. & Bae, Y.-S. (2002). *Life Sci.* **71**, 2145–2152. 10.1016/s0024-3205(02)01997-5 12204772

[bb49] Yukawa, K., Tanaka, T., Bai, T., Li, L., Tsubota, Y., Owada-Makabe, K., Maeda, M., Hoshino, K., Akira, S. & Iso, H. (2006). *Int. J. Mol. Med.* **17**, 869–873. 16596273

